# Single-cell RNA sequencing reveals that MYBL2 in malignant epithelial cells is involved in the development and progression of ovarian cancer

**DOI:** 10.3389/fimmu.2024.1438198

**Published:** 2024-07-29

**Authors:** Wenwen Shao, Zhiheng Lin, Zhikai Xiahou, Fu Zhao, Jue Xu, Xinqi Liu, Pingping Cai

**Affiliations:** ^1^ Shandong University of Traditional Chinese Medicine, Jinan, Shandong, China; ^2^ China Institute of Sport and Health Science, Beijing Sport University, Beijing, China; ^3^ Department of Traditional Chinese Medicine, Shandong Provincial Hospital Affiliated to Shandong First Medical University, Jinan, Shandong, China

**Keywords:** ovarian cancer, neoadjuvant chemotherapy, omentum, epithelial cells, immune microenvironment, single-cell RNA sequencing

## Abstract

**Background:**

Ovarian carcinoma (OC) is a prevalent gynecological malignancy associated with high recurrence rates and mortality, often diagnosed at advanced stages. Despite advances in immunotherapy, immune exhaustion remains a significant challenge in achieving optimal tumor control. However, the exploration of intratumoral heterogeneity of malignant epithelial cells and the ovarian cancer tumor microenvironment is still limited, hindering our comprehensive understanding of the disease.

**Materials and methods:**

Utilizing single-cell RNA sequencing (scRNA-seq), we comprehensively investigated the cellular composition across six ovarian cancer patients with omental metastasis. Our focus centered on analysis of the malignant epithelial cells. Employing CytoTRACE and slingshot pseudotime analyses, we identified critical subpopulations and explored associated transcription factors (TFs) influencing ovarian cancer progression. Furthermore, by integrating clinical factors from a large cohort of bulk RNA sequencing data, we have established a novel prognostic model to investigate the impact of the tumor immune microenvironment on ovarian cancer patients. Furthermore, we have investigated the condition of immunological exhaustion.

**Results:**

Our study identified a distinct and highly proliferative subgroup of malignant epithelial cells, known as C2 TOP2A+ TCs. This subgroup primarily consisted of patients who hadn’t received neoadjuvant chemotherapy. Ovarian cancer patients with elevated TOP2A expression exhibited heightened sensitivity to neoadjuvant chemotherapy (NACT). Moreover, the transcription factor MYBL2 in this subgroup played a critical role in ovarian cancer development. Additionally, we developed an independent prognostic indicator, the TOP2A TCs Risk Score (TTRS), which revealed a correlation between the High TTRS Group and unfavorable outcomes. Furthermore, immune infiltration and drug sensitivity analyses demonstrated increased responsiveness to Paclitaxel, Cisplatin, and Gemcitabine in the Low TTRS Group.

**Conclusion:**

This research deepens our understanding of malignant epithelial cells in ovarian cancer and enhances our knowledge of the ovarian cancer immune microenvironment and immune exhaustion. We have revealed the heightened susceptibility of the C2 TOP2A+ TCs subgroup to neoadjuvant chemotherapy and emphasized the role of MYBL2 within the C2 subgroup in promoting the occurrence and progression of ovarian cancer. These insights provide valuable guidance for the management of ovarian cancer treatment.

## Introduction

Ovarian cancer is one of the deadliest gynecological malignancies, often diagnosed at advanced stages, resulting in poor outcomes (1). Furthermore, around one-third of OC patients experience the development of ascites (2). This disease is characterized by significant heterogeneity, encompassing distinct histological subtypes, molecular profiles, and microenvironmental characteristics that profoundly influence treatment response and clinical outcomes (3). The standard approach in OC management involves surgical intervention combined with platinum-based chemotherapy. In cases of advanced ovarian cancer accompanied by ascites, the combination of surgery and chemotherapy yields a median survival period of 16-22 months (http://seer.cancer.gov/csr/1975_2009_pops09/). However, a subset of ovarian cancer patients develop platinum resistance after initial treatment, and nearly all patients with recurrent disease eventually progress to platinum-resistant ovarian cancer (PROC). Currently, the primary treatment for platinum-resistant ovarian cancer involves non-platinum chemotherapy, which can be used either as a monotherapy or in combination with bevacizumab (4). With advancements in the medical field and pharmaceutical innovation, there has been a gradual reduction in ovarian cancer mortality rates (5). Neoadjuvant chemotherapy (NACT) has emerged as a viable therapeutic option for patients with advanced ovarian cancer who are ineligible for immediate primary tumor debulking surgery (6). NACT provides significant relief from advanced ovarian cancer in terms of optimal debulking surgery (7). However, recent studies have identified a potential risk of platinum resistance associated with NACT (8). Investigating the impact of neoadjuvant chemotherapy on the immune microenvironment of ovarian cancer is crucial for gaining a deeper understanding of tumorigenesis and proliferation mechanisms. Despite advancements in treatment, the overall prognosis remains poor due to the development of chemotherapy resistance and delayed detection, which lead to high recurrence rates. Therefore, it is urgent to further explore and refine therapeutic strategies.

Beyond the primary tumor, omental metastasis assumes comparable importance in the realm of ovarian cancer investigation. Sunila Pradeep and Anil K. Sood, among others, have demonstrated the significance of hematogenous metastasis as a prominent mechanism in ovarian cancer metastasis. Their findings indicate the ability of epithelial ovarian cancer (EOC) cells to undergo hematogenous metastasis to the omentum (9). Epithelial ovarian cancers (EOCs) are recognized as “immunogenic tumors,” as non-spontaneous antitumor immune responses can be observed within tumors, peripheral blood, and ascites of EOC patients (10). Additionally, immune cells from both the tumor and ascites play a crucial role in ovarian cancer.

Over the past two decades, the rapid advancement of immunotherapy has brought about revolutionary changes in the field of cancer treatment. Cancer is associated with immune cell depletion, especially T-cell depletion, which is a state of hypofunction. T cells that remain “un-exhausted” within the tumor microenvironment are often considered key mechanisms targeted by immune checkpoint inhibitors. Previous studies have indicated that T cell exhaustion may be a contributing factor to suboptimal tumor control (11). However, the tumor immune microenvironment of ovarian cancer has not been extensively studied.

Although immune checkpoint inhibition and immunomodulation have shown promise in treating ovarian cancer, their efficacy still lags behind that of numerous other immunogenic tumor types, such as non-small cell lung cancer and melanoma (12, 13). Therefore, it is imperative to explore shared immunological characteristics among ovarian cancer patients to identify optimal indicators.

Single-cell sequencing techniques offer valuable insights into various diseases, including cancer biology (14). Single-cell RNA sequencing (scRNA-seq) has gained traction in exploring cellular heterogeneity in multiple tumor tissues, such as melanoma (15, 16), glioblastoma (17), and clear cell renal cell carcinomas (18), and non-neoplastic diseases like central nervous system (CNS) diseases (19). However, the application of scRNA-seq in ovarian cancer clinical samples remains limited.

In this study, we employed single-cell sequencing to investigate cells obtained from the omentum of six individuals with ovarian cancer. Specifically, our focus was on examining malignant epithelial cells characterized by heightened copy number variation (CNV) levels. We aimed to unravel the intricacies within diverse cell populations, explore their developmental trajectories, identify functional enrichments, and analyze the associated transcription factors. Novel prognostic models were constructed using sophisticated bioinformatics tools like ESTIMATE, CIBERSORT, and Xcell to uncover the immune microenvironment of ovarian cancer. Our primary objective was to discover fresh biomarkers and gain a deeper understanding of the complex interplay between epithelial cells in peritoneal metastases and ovarian cancer, ultimately providing novel insights for the treatment of this disease.

## Method

### Acquisition of ovarian cancer data

Ovarian cancer data obtained through single-cell RNA sequencing (scRNA-seq) was retrieved from the NCBI Gene Expression Omnibus (GEO) database (https://www.ncbi.nlm.nih.gov/geo/). The dataset used for single-cell analysis consisted of omentum samples from six ovarian cancer patients, with the accession number GSE147082 (GSM4416534, GSM4416535, GSM4416536, GSM4416537, GSM4416538 and GSM4416539). The database provided detailed data on 6 ovarian cancer patients with metastatic omental tumors, including their age, race, origin of disease, histologic type, histological grade, and neoadjuvant therapy. For further details, refer to **Supplementary Table 1**. Bulk RNA-seq data sets and clinical data were obtained from the Cancer Genome Atlas (TCGA) (https://portal.gdc.cancer.gov/). Genetic mutation data and clinical information, such as survival details, were obtainable from this database for ovarian cancer patients.

### Quality control and dimensionality reduction clustering

The raw data underwent processing using the “Seurat” package (version 4.3.0) (20). The “DoubletFinder” package (21) was applied to identify and eliminate double peaks, while the “PercentageFeatureSet” function was used to enhance cellular quality and exclude cells of low quality based on the following criteria (1): 300 < total number of genes detected in a single cell (nFeature) < 8000, (2) 500 < total transcriptomic count in a single cell (nCount) < 50,000. (3) The percentage of mitochondrial genes expressed in a single cell was less than 20%, and (4) the expression of erythrocyte genes in a single cell was below 5%.

The data was normalized using the “NormalizeData” function. After obtaining high-quality cells, we selected the top 2,000 genes exhibiting significant variability (22), and gene expression was processed using ScaleData. Dimensionality reduction was performed by extracting the first 30 principal components (PCs), and a corresponding Uniform Manifold Approximation and Projection (UMAP) was generated (23–25). And the data followed by the application of the “harmony” R package (version 0.1.0) to mitigate batch effects. Cell clusters were identified based on the expression of established classical cell marker genes.

### Screening and subgroup identification of malignant epithelial cells in ovarian cancer

To distinguish between non-malignant and malignant cells within the ovarian cancer epithelial cell context, we utilized the “InferCNV” tool (https://github.com/broadinstitute/inferCNV/wiki) to infer copy number variation (CNV) in distinct cell subsets. Specifically, epithelial cells (ECs) were used as the reference group for inferCNV, with elevated CNV levels indicating tumor-derived epithelial cells.

Epithelial cells from ovarian cancer tumors were analyzed, the data was normalized and scaled using “NormalizeData”, selecting the top 2000 genes with high variability and performing similar steps as before, and further processed the data using “ScaleData” (15, 16). PCA was then conducted on the initial 30 PCs of the single-cell data, followed by batch effect removal using the ‘harmony’ R package (version 0.1.0). Cluster analysis was performed using “FindNeighbors” and “FindClusters” in Seurat, and subgroups were annotated based on characteristic marker genes. Finally, UMAP plots were generated to visualize the distinct cell subgroups (17, 18).

### Heterogeneity of ovarian cancer subsets

The “FindAllMarkers” function was employed to identify genes with differential expression (DEGs) in each subset, which were subsequently analyzed for enrichment in Gene Ontology Biological Processes (GO-BP) using the “ClusterProfiler” R package (version 0.1.1) for GO-BP enrichment analysis (26–28). Gene functions in various subgroups were examined and ranked using Gene Set Enrichment Analysis (GSEA) (29) with the c2.cp.kegg.v7.5.1.symbols.gmt dataset.

### Stemness analysis and trajectory analysis of subpopulations

To explore the varying degrees of differentiation among distinct subgroups of ovarian cancer, we employed CytoTRACE analysis (30) to obtain the CytoTRACE score for each subgroup, allowing us to infer their respective differentiation states. The “Slingshot” package (version 2.6.0) (31) was utilized to infer the developmental trajectory of each subpopulation, while the “getlineage” and “getCurves” functions facilitated the inference of differentiation trajectory and evaluation of expression levels over time for each ovarian cancer subpopulation.

### Transcription factor analysis

We employed the pySCENIC algorithm to explore the transcription factors and regulators (TFs) within each subgroup. Initially, GRNBoost was applied to establish the relationships between transcription factors (TFs) and target genes, followed by DNA motif analysis to identify potential direct binding targets. AUcell was then utilized to evaluate the activity of each regulator within the cells, and the top 5 TFs with the most elevated scores were selected.

### Cellchat analysis

To investigate the intricate cell-to-cell communication among the various subgroups, we employed the “cellchat” package (version 1.6.1) (32) to assess intercellular interactions between the subgroups, focusing on signal pathways and receptor-ligand interactions.

### Construction of a novel prognostic risk model and its validation

Key subgroups were subjected to univariate Cox analysis and LASSO Cox regression (33–36) in the TCGA cohort. Subsequently, prognostic genes were identified to construct a prognostic risk model, and the Risk score was determined through multivariate Cox analysis. The specific formula used was: Risk Score= $\sum_{i}^{\text{n}}\text{Xi}\times \text{Yi}$ (X: coefficient, Y: gene expression level). Samples were then divided into high-risk score and low-risk score groups based on the median. Kaplan-Meier (K-M) curves and Receiver Operating Characteristic (ROC) curves (37–39) were generated to validate the prognostic value of the model.

Furthermore, a nomogram was constructed to predict patient prognosis by incorporating the Risk Score and clinical factors (40). The performance of the nomogram was assessed using ROC curves and the concordance index (C-index). Additionally, the correlation between prognosis-related genes and the Risk Score was investigated.

### Immune infiltration analysis and functional enrichment analysis

We utilized CIBERSORT and the Xcell algorithm to analyze immune infiltration in the high and low scoring groups, uncovering correlations between immune infiltrating cells and prognosis-related genes (41–43). Furthermore, we evaluated the Tumor Immune Dysfunction and Exclusion (TIDE) scores for both high- and low-scoring groups, and compared the expression levels of genes associated with immune checkpoints between the high- and low-risk groups using the Wilcoxon test (44).

To explore the heterogeneity of the high and low scoring groups, we used the “DESeq2” package to identify differentially expressed genes (DEGs). Next, we employed the “clusterProfiler” R package (version 4.6.2) (45) to analyze Kyoto Encyclopedia of Genes and Genomes (KEGG) pathways and perform enrichment analyses on Gene Ontology Biological Processes (GOBP), Gene Ontology Cellular Components (GOCC), and Gene Ontology Molecular Functions (GOMF) (46–48).

### Gene mutation data

Ovarian cancer mutation data was obtained from the TCGA database, and different Tumor Mutation Burden (TMB) groups were assessed using the “maftools” R package (49, 50). The relationship between the score and TMB was analyzed using a Spearman correlation test. Based on the median TMB, specimens were categorized into high and low TMB groups, and Kaplan-Meier survival analysis was conducted to examine prognostic differences between the groups.

### Assessment of drug sensitivity

The half-maximum inhibitory concentration (IC50) of different chemotherapy drug groups was evaluated using the “pRRophetic” R package (version 0.5) (51).

### Cell lines and cultures

The SK-OV-3 and A2780 cell lines were obtained from the American Type Culture Collection (ATCC). SK-OV-3 cells were cultured in McCoy’s 5A medium, while A2780 cells were cultured in PRMI 1640 medium, both supplemented with 10% fetal bovine serum (Gibco BRL, USA) and 1% streptomycin/penicillin. Cultures were maintained at 37°C with 5% CO2 and 95% humidity.

### Transfection

MYBL2 knockdown was achieved using siRNA constructs obtained from GenePharma in Suzhou, China. Transfection followed the specific instructions of Lipofectamine 3000RNAiMAX (Invitrogen, USA), including the introduction of negative control (si-NC) and knockdown (si-MYBL2-1 and si-MYBL2-2).

### Colony formation

Transfected cells (1×10^3^ per well) were plated in 6-well plates for colony formation assays and incubated for 2 weeks. Subsequently, the cells were subjected to sequential fixation (4% paraformaldehyde) and staining (Crystal Violet), followed by photography and counting analysis.

### Cell viability assay

Transfected SK-OV-3 and A2780 cells were evaluated for viability with the CCK-8 test. Cells were placed in 96-well plates with a concentration of 5 × 10^3^ cells per well and incubated for 24 hours. Next, each well received 10 mL of CCK-8 reagent (A311-01, Vazyme) and was then incubated at 37°C in the dark for 2 hours. OD values were measured at 450 nm on days 1, 2, 3, and 4, and a line graph was created using the recorded data.

### Transwell

Transwell chambers were utilized to assess the migration and invasion capacities of cells. Transwell chambers were either coated with (for transwell invasion assay) or left uncoated (for transwell migration assay) matrix gel (BD Biosciences, USA). The upper chamber used with serum-free medium, and the lower chamber contained medium supplemented with serum. Following a 48-hour incubation period in a cell culture incubator, the cells were treated with 4% paraformaldehyde and then stained using crystal violet. Subsequently, cell counting was performed using a microscope to observe the invasive and migratory capabilities.

### Wound healing assay

Transfected cells were seeded and cultured in 6-well plates. At around 95% cell density, a sterile 200- μL pipette was utilized to make a linear scratch on the cell layer. The plate was gently rinsed with PBS to remove non-adherent cells and debris. Then, the culture medium was replaced to support cell growth. Images of the scrape were captured at both 0 and 48 hours from an identical angle, and the breadth of the scrape was assessed.

### Statistical analysis

R software (version 4.3.0) and Python software (version 4.2.0) were used for statistical analysis. The significance of variations among different groups was assessed using the Wilcoxon test and Pearson correlation coefficients. Significance levels were denoted as follows: *P < 0.05, **P < 0.01, ***P < 0.001, and ****P < 0.0001. “ns” was used to indicate a lack of significant difference.

## Results

### Identification of main cell types in ovarian cancer

**Figure 1:**
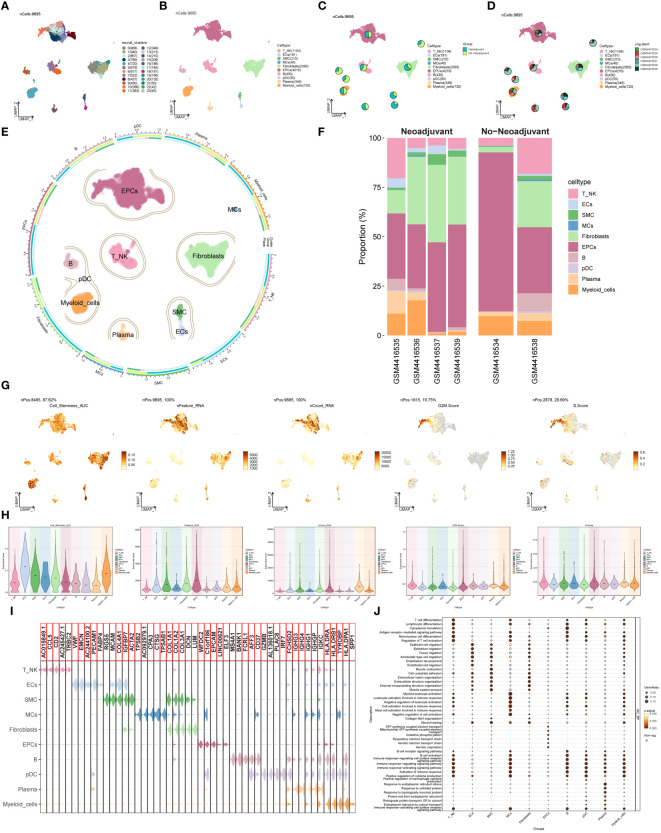
Main cell types of ovarian cancer. **(A)** UMAP visualization exhibited 24 distinct seurat clusters comprising 9,695 high-quality cells from ovarian cancer. **(B)** UMAP plot showcasing the distribution of 10 cell types. **(C, D)** UMAP plots combined with pie charts illustrating tissue types (Neoadjuvant and No−Neoadjuvant) and sample sources for each cell type. **(E)** Comprehensive UMAP plot displaying the distribution of each cell type, along with its cell cycle and tissue type ratio. **(F)** Bar graph demonstrating sample sources and the proportion of cell types in two tissue types (Neoadjuvant and No−Neoadjuvant). **(G, H)** UMAP and violin plots revealing the Cell_Stemness_AUC, nFeature_RNA, nCount_RNA, G2M.Score, and S.Score for each cell type, respectively. **(I)** Violin plot displaying the top 5 marker genes of each cell type. **(J)** Bubble chart presenting the results of GOBP enrichment analysis for DEGs from diverse cell types.

Single-cell data was collected from six patients diagnosed with advanced ovarian cancer. After quality assessment and removal of batch discrepancies, we obtained a total of 9,695 cells of exceptional quality. These cells were then classified into 24 seurat groups and visualized using the Uniform Manifold Approximation and Projection (UMAP) chart (**Figure 1A**). Based on their distinctive gene expression profiles, the groups were assigned to 10 different cell types: T and NK Cells (T_NK), Endothelial Cells (ECs), Smooth Muscle Cells (SMC), Mast Cells (MCs), Fibroblasts, Endothelial Progenitor Cells (EPCs), B Cells (B), Plasmacytoid Dendritic Cells (pDC), Plasma Cells (Plasma), and Myeloid Cells (**Figure 1B**). Additionally, we categorized the cells into two tissue types, Neoadjuvant and No−Neoadjuvant, based on whether the patients received neoadjuvant chemotherapy (NACT). The distribution of tissue types for each cell type was shown in **Figure 1C**, and the origin of samples for each cell type was illustrated in **Figure 1D**. The UMAP plot provided a comprehensive visualization of the distribution of the 10 cell types, their tissue sources, and the proportion of cell cycles (**Figure 1E**). Among the six patients, samples GSM4416534 and GSM4416538 did not receive neoadjuvant chemotherapy (**Figure 1F**).

To evaluate the characteristics of individual cell types, we computed the Cell Stemness AUC, nFeature RNA, nCount RNA, G2M.Score, and S.Score. The findings were presented using UMAP and violin plots (**Figures 1G, H**). The results indicated that ECs, Fibroblasts, and Myeloid cells exhibited higher Cell Stemness AUC values, while EPCs and Myeloid cells displayed higher G2M scores. Violin plots in **Figure 1I** highlighted the top 5 marker genes for each cell type. The DEGs of cell types were used to analyze Gene Ontology Biological Processes (GOBP), and the findings were displayed in a bubble plot (**Figure 1J**).

### Identification and analysis of tumor epithelial cell subsets

**Figure 2:**
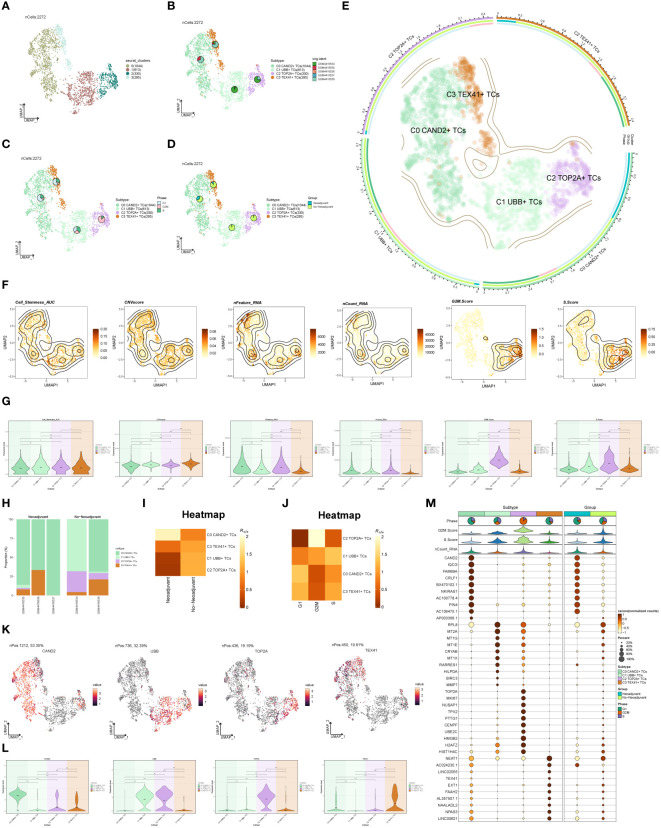
Subgroup identification of ovarian cancer. **(A)** UMAP visualization showing the arrangement of four distinct seurat clusters within ovarian cancer epithelial cells. **(B-D)** UMAP plots and pie charts displaying the origins of samples, cell cycle stages (G1, G2M, and S), and various tissue categories (Neoadjuvant and No−Neoadjuvant) within the four subgroups. **(E)** A comprehensive UMAP plot illustrating the distribution of each sub-cluster, along with its cell cycle ratio and tissue type ratio. **(F, G)** Cell_Stemness_AUC, CNVScore, nFeature_RNA, nCount_RNA, G2M.Score, and S.Score of each subgroup displayed in UMAP plots and violin plots. **(H)** Bar graphs illustrating the subgroup proportion and tissue types of different samples. **(I, J)** Heatmaps showing the tissue types and cell cycle preferences of the four subgroups, respectively. **(K, L)** Distribution of named genes for the four subgroups visualized using UMAP plots and violin plots for each subgroup. **(M)** Bubble chart displaying the top 10 marker genes for each subgroup, along with their expression levels in various tissue types. *P < 0.05; **P < 0.01; ***P < 0.001; and ****P < 0.0001; ns indicated no significant difference.

To distinguish malignant cells from non-malignant cells in ovarian cancer epithelial cells, we employed InferCNV and defined cells with high-level CNV as tumor cells (**Supplementary Figure S1**). After screening and quality control, we obtained a total of 2,272 tumor epithelial cells. Through dimensionality reduction cluster analysis, these cells were classified into 4 seurat clusters (**Figure 2A**). Based on marker genes, they were named C0 CAND2+ TCs (1044), C1 UBB+ TCs (613), C2 TOP2A+ TCs (330), and C3 TEX41+ TCs (285), with a visualization created using UMAP plots (**Figure 2B**). These epithelial tumor cells originated from five different samples (GSM4416534, GSM4416535, GSM4416536, GSM4416537, GSM4416538). The cell cycles (G1, G2M, and S) and tissue types (Neoadjuvant and No-Neoadjuvant) for the four subgroups were displayed using UMAP plots combined with pie charts. (**Figures 2C, D**). Most C2 TOP2A+ TCs were in the G2M phase and did not receive neoadjuvant chemotherapy. A comprehensive view of the subpopulation distribution, cell cycle distribution, and tissue types was depicted in **Figure 2E**.

The Cell_Stemness_AUC, CNVScore, nFeature_RNA, nCount_RNA, G2M.Score, and S.Score were calculated for each subgroup to assess their characteristics. These results were visualized using UMAP and violin plots (**Figures 2F, G**). The findings revealed that C2 TOP2A+ TCs had higher values for nFeature_RNA, G2M.Score, and nCount_RNA. The subgroup proportion and tissue types of different samples were analyzed (**Figure 2H**), demonstrating that the majority of C2 TOP2A+ TCs originated from samples GSM4416534 and GSM4416538, which did not receive neoadjuvant chemotherapy. We estimated the tissue and cell cycle preferences of each subgroup using the Ro/e value and presented the analysis results as heatmaps (**Figures 2I, J**). Consistent with previous results, C0 CAND2+ TCs preferred neoadjuvant treatment, while C2 TOP2A+ TCs showed a preference for non-neoadjuvant treatment. We also analyzed the distribution of named genes within the four subgroups (**Figures 2K, L**) and observed that UBB, the named gene of the C1 subgroup, was also expressed in the C2 subgroup, while TOP2A, the named gene of the C2 subgroup, was almost exclusively expressed in the C2 subgroup. To showcase the top 10 marker genes in each subgroup, bubble plots were employed and the distribution across different tissue types (Neoadjuvant and No−Neoadjuvant) was depicted (**Figure 2M**).

### DEGs and enrichment analysis of distinct subgroups

**Figure 3:**
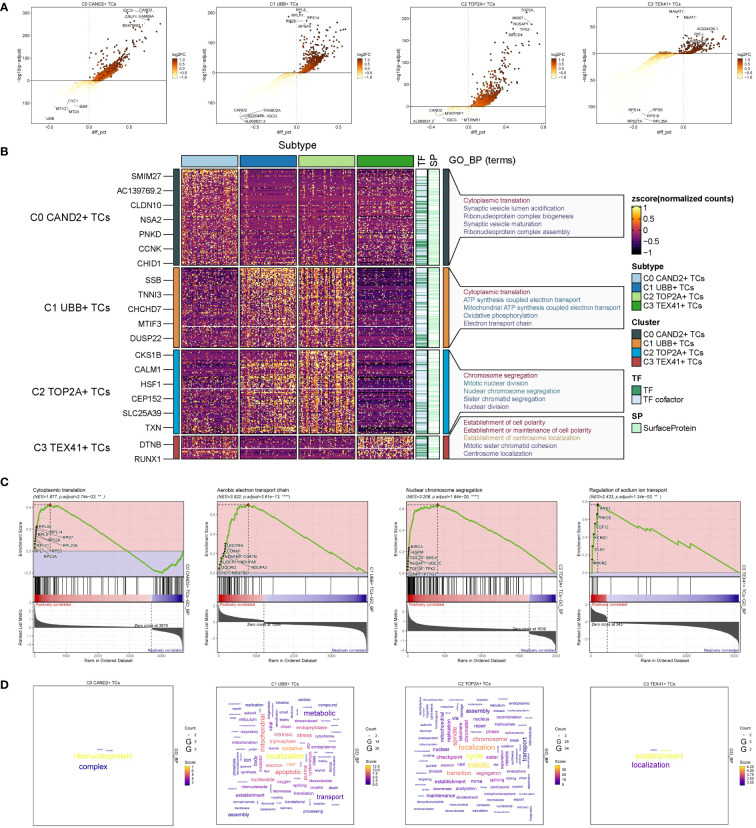
Identification and enrichment analysis of subgroup DEGs in ovarian cancer. **(A)** Volcano plots presenting DEGs of four cell subsets. **(B)** Heatmap displaying the Gene Ontology Biological Process enrichment terms of Differentially Expressed Genes in four distinct cell populations. **(C)** Results of GSEA enrichment analysis on four cell subsets. **(D)** Cloud charts displaying the outcomes of GO-BP enrichment analysis based on the gene count.

To explore the diversity within each ovarian cancer subgroup, we examined the differentially expressed genes (DEGs) in the four subgroups and visualized the top 5 up-regulated and down-regulated genes in volcano plots (**Figure 3A**). To investigate the associated biological processes for each subgroup, we conducted Gene Ontology Biological Processes (GOBP) analysis, presenting the findings in **Figure 3B**. C0 CAND2+ TCs were associated with various biological processes, including cytoplasmic translation, synaptic vesicle lumen acidification, ribonucleoprotein complex biogenesis, synaptic vesicle maturation, and ribonucleoprotein complex assembly, among others. The DEGs in C1 UBB+ tumor cells were enriched in cytoplasmic translation, ATP synthesis coupled electron transport, mitochondrial ATP synthesis coupled electron transport, oxidative phosphorylation, and the electron transport chain. C2 TOP2A+ TCs were associated with chromosome segregation, mitotic nuclear division, nuclear chromosome segregation, sister chromatid segregation, and nuclear division. C3 TEX41+ TCs were involved in establishment of cell polarity, establishment or maintenance of cell polarity, establishment of centrosome localization, mitotic sister chromatid cohesion, centrosome localization, and related pathways.

GSEA enrichment analysis was performed on four subgroups, and the maximum value of NES (normalized enrichment score) in each subgroup was selected for display (**Figure 3C**). The findings indicated that the cytoplasmic translation pathway of C0 CAND2 + TCs was cytoplasmic translation (NES = 1.877), and C1 UBB+ TCs was enriched in the aerobic electron transport chain (NES = 3.522). C2 TOP2A+ TCs was closely related to the nuclear chromosome segregation (NES = 3.206), while C3 TEX41+ TCs was enriched regulation of sodium ion transport (NES = 2.433).

Based on the expression count of genes within each subgroup, we conducted GOBP enrichment analysis on highly expressed genes and visualized the results using cloud diagrams based on the enrichment scores (**Figure 3D**). C0 CAND2+ TCs were associated with ribonucleoprotein processes, C1 UBB+ TCs exhibited significant enrichment in localization and oxidative processes, C2 TOP2A+ TCs were enriched in localization, mitotic, and transition processes, while C3 TEX41+ TCs were linked to establishment and localization processes.

### Stemness analysis and trajectory analysis of different subgroups

**Figure 4:**
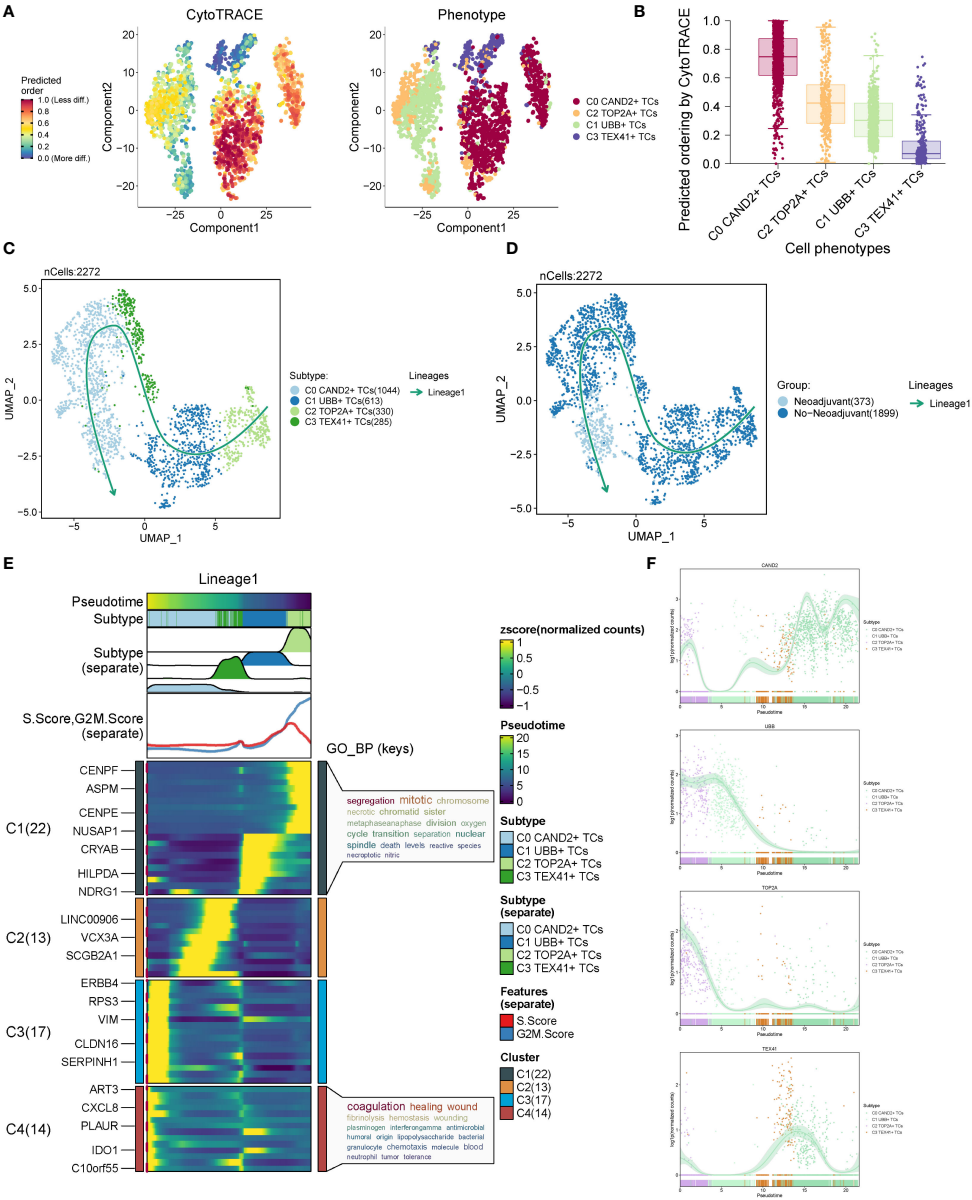
CytoTRACE analysis and pseudotime analysis of cell subsets. **(A)** CytoTRACE analysis results of four cell subsets. In the left panel, dark green indicated greater differentiation (low stemness), while dark red indicated less differentiation (high stemness). In the right panel, different colors represented different ovarian cancer subgroups. **(B)** CytoTRACE scores for four cell subsets were displayed. **(C)** UMAP plot presenting the results of slingshot pseudotime analysis of four cell subsets. The specific pseudotime trajectories of the four cell subsets were C2 TOP2A+ TCs → C1 UBB+ TCs → C3 TEX41+ TCs → C0 CAND2+ TCs, constituting one lineage in total. **(D)** UMAP plots showing the pseudotime trajectory of different tissue types: No−Neoadjuvant →Neoadjuvant. **(E)** Heatmap displaying the changes of DEGs in each subset with pseudotime and the results of GO-BP enrichment analysis. **(F)** Scatter plots exhibiting the changing trend of named genes in four subgroups with pseudotime.

To assess the stemness properties and evaluate the developmental trajectory of the four subgroups, we conducted CytoTRACE analysis and slingshot pseudotime analysis. The results from CytoTRACE analysis revealed that the C0 CAND2+ TCs and C2 TOP2A+ TCs exhibited higher cytotrace scores, indicating their greater differentiation potential, while the C3 TEX41+ TCs showed the lowest cytotrace scores, suggesting they might be at the terminal stage of differentiation (**Figures 4A, B**). Slingshot pseudotime analysis was performed on the four subgroups, and the UMAP diagram visualized the progression from C2 TOP2A+ TCs to C1 UBB+ TCs, then to C3 TEX41+ TCs, and finally to C0 CAND2+ TCs, which closely aligned with the CytoTRACE analysis results (**Figure 4C**). The distribution trajectories of different tissue types with Lineage1 were shown in **Figure 4D**, indicating that the transition paths for tissue types were from No−Neoadjuvant to Neoadjuvant. The distribution of differentially expressed genes (DEGs) within the four subgroups with slingshot pseudotime analysis in Lineage1 was also analyzed (**Figure 4E**), and these DEGs were further examined using gene ontology biological processes (GOBP) enrichment analysis. The DEGs in the C0 subgroup were enriched in segregation mitotic and other signaling pathways, while the DEGs in the C3 subgroup were enriched in coagulation healing wound and other pathways.

The distribution patterns of named genes within the four subgroups along the trajectory of slingshot pseudotime analysis were illustrated in **Figure 4F**. As the pseudotime progressed, the expression of the C0 subgroup’s named gene, CAND2, gradually increased. The named gene UBB of the C1 subgroup and the named gene TOP2A of the C2 subgroup exhibited higher expression in Lineage1 initially and gradually declined. In contrast, the expression of the named gene TEX41 of the C3 subgroup showed an inclination to increase and then decrease in the pseudotime trajectory.

Based on the above analysis, the C2 TOP2A+ TCs subgroup of malignant epithelial cells mainly originated from patients who had not received neoadjuvant chemotherapy. CytoTRACE and slingshot pseudotime analyses indicated that C2 TOP2A+ TCs was at the initial stage of differentiation with high differentiation potential. Therefore, it was speculated that this subgroup was highly sensitive to neoadjuvant chemotherapy, which could induce its transition to a subgroup with lower proliferative capacity.

### Analysis of gene regulatory networks in different subgroups

**Figure 5:**
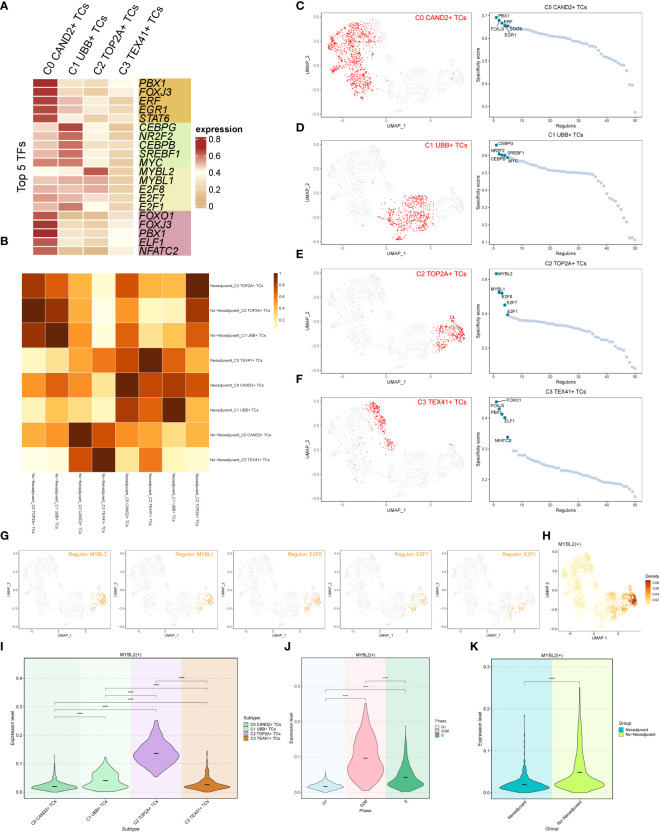
Transcription factor (TF) analysis of ovarian cancer subgroups. **(A)** Heatmap displaying the top 5 transcription factors (TFs) of the four subgroups. **(B)** Heatmap illustrating the correlation between two tissue types (Neoadjuvant and No−Neoadjuvant) of the four subgroups. **(C-F)** UMAP plots and scatter plots showcasing the TF ranking of each subgroup and its distribution, respectively. **(G)** UMAP plot displaying the distribution of the C2 subgroup’s top 5 TFs in each subgroup. **(H)** Density distribution of the C2 subgroup Top 1 TF (MYBL2). **(I-K)** Violin plots exhibiting the expression level of MYBL2 in each subgroup **(I)**, each cell cycle **(J)**, and each tissue type **(K)**, respectively.

We employed pySCENIC to infer the gene regulatory networks within ovarian cancer cell subgroups. The heatmap visualized the top 5 transcription factors (TFs) within each cell subgroup (**Figure 5A**). The most active TFs in each subgroup were identified as PBX1 (C0 CAND2+ TCs), CEBPG (C1 UBB+ TCs), MYBL2 (C2 TOP2A+ TCs), and FOXO1 (C3 TEX41+ TCs). Based on whether the subgroups received neoadjuvant treatment, we studied the relationships among various tissue types, and the corresponding findings were displayed in **Figure 5B**.

The rankings of the top 5 TFs within each subgroup were shown in **Figures 5C-F**, and their specific distributions were visualized using UMAP and scatter plots. In particular, the C2 TOP2A+ TCs subgroup had the following top 5 TFs: MYBL2, MYBL1, E2F8, E2F7, and E2F1. The UMAP plots depicted the distribution of these five TFs in in each subgroup (**Figure 5G**). The results indicated that MYBL2, MYBL1, and E2F8 were primarily distributed within the C2 TOP2A+ TCs subgroup, while E2F7 and E2F1 were also observed in the C1 UBB+ TCs subgroup. The density distribution of the top 1 TF (MYBL2) within the C2 subgroup was illustrated in **Figure 5H**. Furthermore, violin plots were utilized to demonstrate the expression distribution of MYBL2 in different subgroups, cell cycle phases, and tissue types (**Figures 5I-K**), with statistically significant differences observed. Notably, MYBL2 TF was predominantly expressed in the C2 TOP2A+ TCs subgroup. It exhibited the highest expression level during the G2M phase of the cell cycle, and its expression was higher in tissue types without neoadjuvant treatment compared to those that underwent neoadjuvant treatment. In summary, based on the previous analyses, it was observed that the expression of C2 TOP2A+ TCs and its top 1 TF (MYBL2) decreased following neoadjuvant chemotherapy.

The top TF MYBL2 in the C2 TOP2A+ TCs subgroup is closely linked to tumorigenesis and progression, with studies indicating its association with poor prognosis in different tumor (52–55).

### Cellchat analysis

**Figure 6:**
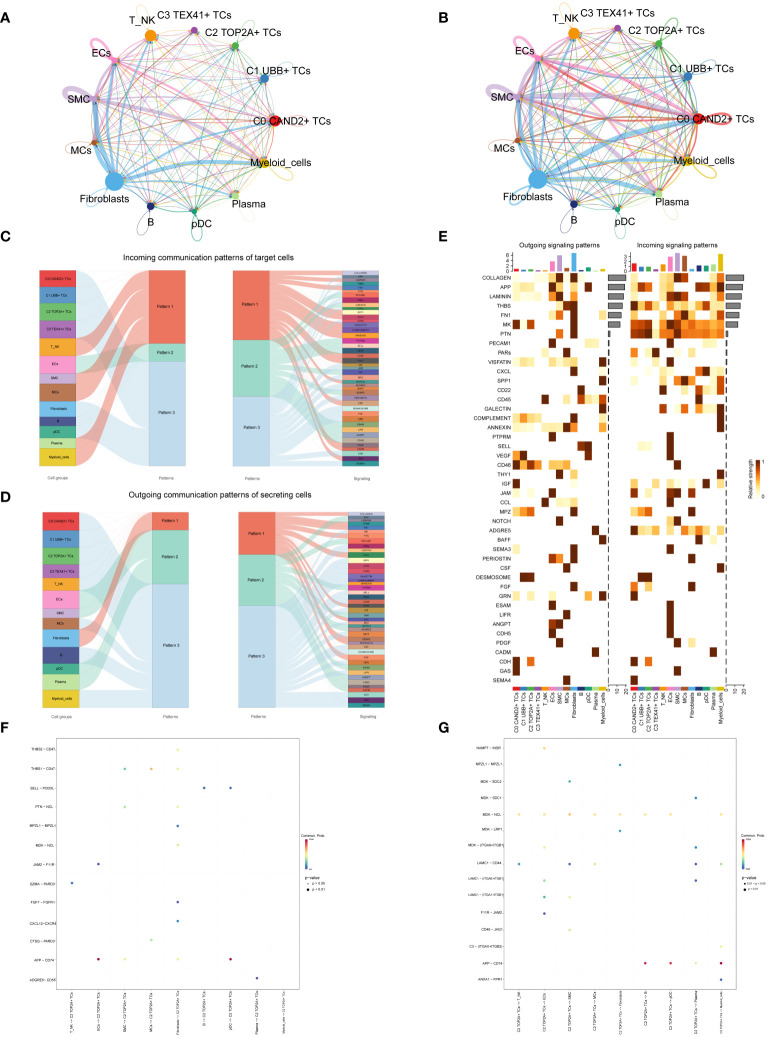
Subgroup interaction analysis. **(A, B)** Circle plots displaying the intensity **(A)** and number **(B)** of interactions between large groups and subgroups of ovarian cancer. The thicker the line between the two cell types, the greater the strength or quantity of the interaction. **(C)** Sankey diagrams presenting the deduced incoming communication patterns of target cells. **(D)** Sankey charts illustrating the deduced outward communication patterns in secreting cells. **(E)** Heatmap revealing outgoing and incoming signaling patterns for all subgroups. **(F, G)** Dot plots showing the receptor-ligand pairs of the C2 subgroup and other subgroups, along with their interaction intensity.

Cell communication within large and small groups of ovarian cancer was examined, with circle plots used to visualize the intensity (**Figure 6A**) and quantity (**Figure 6B**) of interactions among various cell types using circle plots.

Through cellchat analysis, we determined the corresponding incoming communication patterns (**Figure 6C**) and outgoing communication patterns (**Figure 6D**) to reveal the potential communication network between C2 subgroup and other subgroups, and speculated three incoming signal modes and outgoing signal modes. Analysis of expected communication patterns indicated that fibroblasts, B cells, pDCs and 4 subgroups of malignant epithelial cells were associated with Pattern 3. The signaling pathways related to Pattern 3 included IGF, CD45, FGF, DESMOSOME, CDH, etc. The inferred outgoing communication patterns showed that four subgroups, T NK, MCs, B cells, and myeloid cells, were all characterized by Pattern 3, including MK, PARs, SPP1, CD45, and other signal pathways.

To visualize the intensity of incoming and outgoing signals in the interaction between all subgroups, we generated a heatmap (**Figure 6E**). For the putative incoming signaling patterns, most ovarian cancer subgroups displayed associations with the MK signal pathway. Concerning the outgoing signaling patterns, the primary target cells involved were the C0 and C2 subgroups.

Examination of receptor-ligand connections between the C2 subgroup and different subgroups revealed that the association between APP and CD74 was more pronounced when the C2 subgroup served as the target (**Figure 6F**). When the C2 subgroup served as the source, MDK-NCL and APP-CD74 exhibited stronger associations with other subpopulations (**Figure 6G**).

### Analysis related to the partial exhaustion pathway

**Figure 7:**
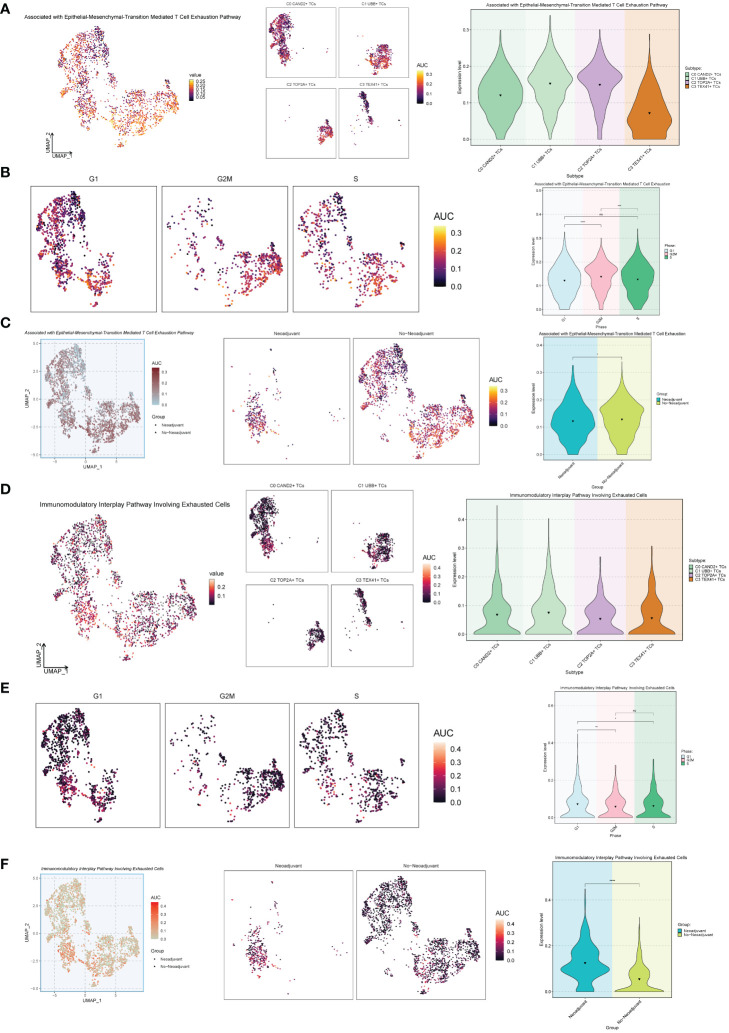
Exhaustion pathway analysis. **(A)** The UMAP and violin plots respectively displayed the expression of different subtypes of malignant ovarian cancer epithelial cells in the Associated with Epithelial-Mesenchymal-Transition Mediated T Cell Exhaustion Pathway. **(B)** The UMAP and violin plots respectively displayed the expression of different phases of malignant ovarian cancer epithelial cells in the Associated with Epithelial-Mesenchymal-Transition Mediated T Cell Exhaustion Pathway. **(C)** The UMAP and violin plots respectively displayed the expression of different groups of malignant ovarian cancer epithelial cells in the Associated with Epithelial-Mesenchymal-Transition Mediated T Cell Exhaustion Pathway. **(D–F)** The UMAP and violin plots respectively displayed the expression of different subtypes **(D)**, different phases **(E)**, and different groups **(F)** of malignant ovarian cancer epithelial cells in the Immunomodulatory Interplay Pathway Involving Exhausted Cells. *P < 0.05; **P < 0.01; ***P < 0.001; and ****P < 0.0001; ns indicated no significant difference.

Next, we conducted an exhaustion pathway analysis on malignant epithelial cells. Based on previous literature, we examined the expression of Associated with Epithelial-Mesenchymal-Transition Mediated T Cell Exhaustion Pathway as well as the Immunomodulatory Interplay Pathway Involving Exhausted Cells in malignant epithelial cells, as shown in **Figure 7**. The results indicated that the C2 TOP2A+ TCs and C1 UBB+ TCs subgroups, G2M Phase, and No−Neoadjuvant had relatively high expression levels in the Associated with Epithelial-Mesenchymal-Transition Mediated T Cell Exhaustion Pathway pathways (**Figures 7A-C**). In contrast, for the Immunomodulatory Interplay Pathway Involving Exhausted Cells, the C1 UBB+ TCs subgroup, G1 Phase, and Neoadjuvant showed higher expression levels (**Figures 7D-F**).

**Figure 8:**
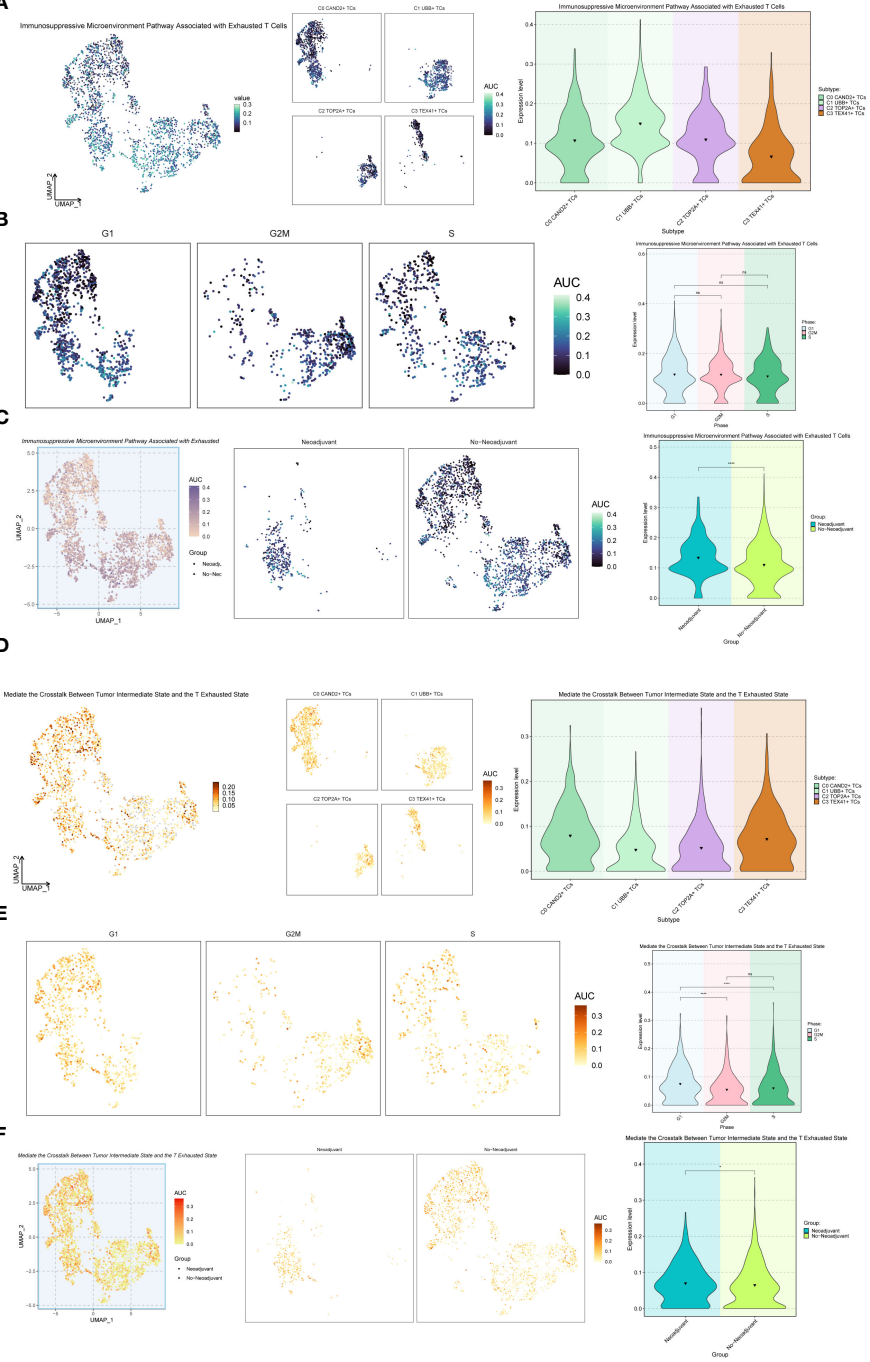
Further analysis of the exhaustion pathway. **(A-C)** UMAP plots and violin plots respectively showed the expression of the Immunosuppressive Microenvironment Pathway Associated with Exhausted T Cells pathway in different subtypes **(A)**, phases **(B)**, and groups **(C)** of malignant ovarian epithelial cells. **(D–F)** UMAP plots and violin plots respectively showed the expression of the pathway Mediate the Crosstalk Between Tumor Intermediate State and the T Exhausted State in different subtypes **(D)**, phases **(E)**, and groups **(F)** of malignant ovarian epithelial cells. *P < 0.05 and ****P < 0.0001; ns indicated no significant difference.

Additionally, we explored the Immunosuppressive Microenvironment Pathway Associated with Exhausted T Cells and the Mediate the Crosstalk Between Tumor Intermediate State and the T Exhausted State pathway (**Figure 8**). For the Immunosuppressive Microenvironment Pathway Associated with Exhausted T Cells pathway, the C1 UBB+ TCs subgroup and Neoadjuvant exhibited higher expression levels (**Figures 8A-C**). However, for the Mediate the Crosstalk Between Tumor Intermediate State and the T Exhausted State pathway, the C0 CAND2+ TCs and C3 TEX41+ TCs, G1 Phase, and Neoadjuvant had higher expression levels (**Figures 8D-F**).

### Establishment of prognostic model and correlation analysis

**Figure 9:**
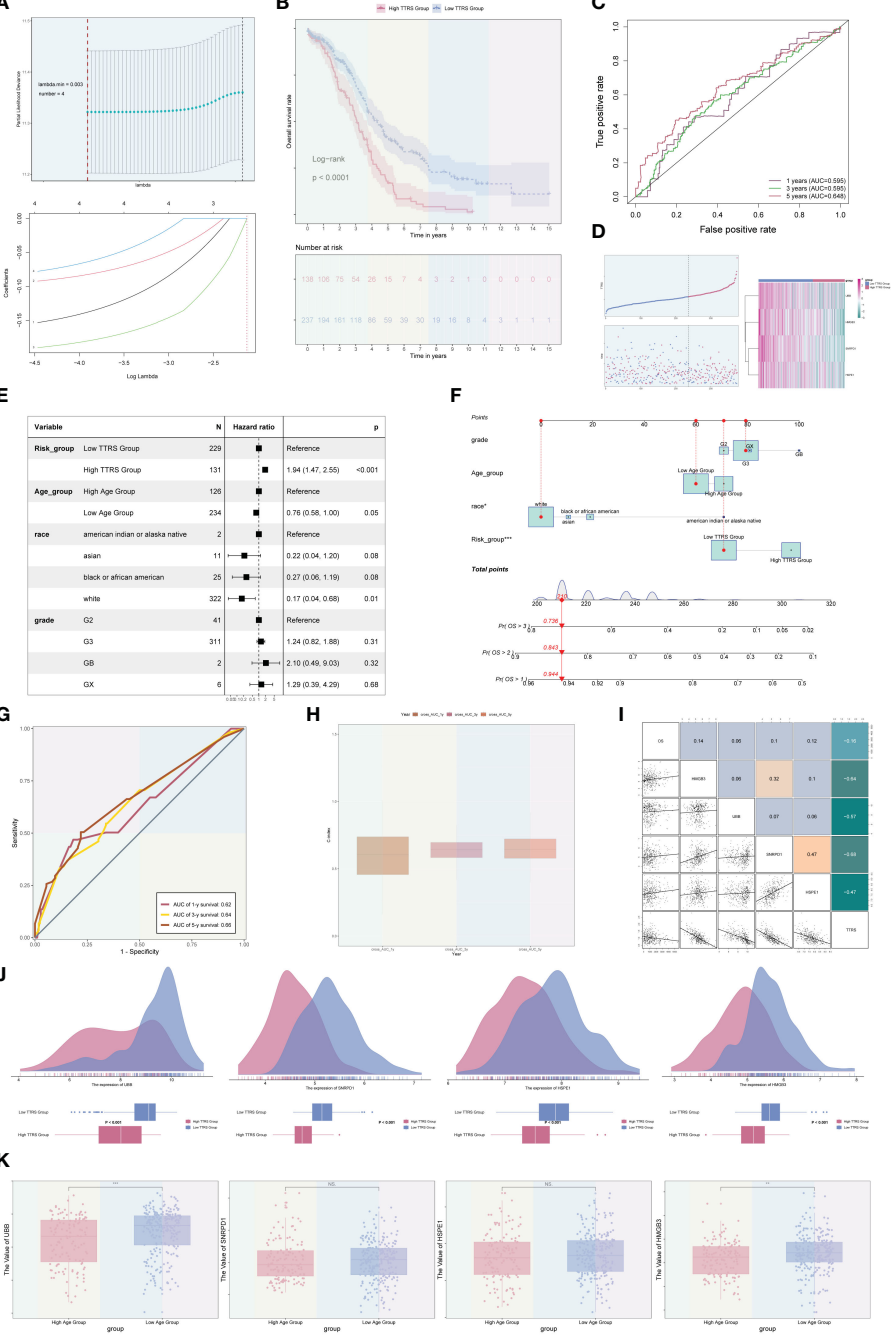
Development and correlation analysis of the TOP2A TCs Risk Score (TTRS). **(A)** LASSO regression analysis yielding optimal results with a lambda.min value of 0.003. **(B)** Kaplan-Meier survival curve of the high TOP2A TCs Risk Score (TTRS) group and the low TTRS group. **(C)** ROC curves displaying the AUCs for 1, 3, and 5-year intervals. **(D)** Scatter plots and curve plots illustrating the survival state of high and low TTRS groups over time and the situation of TOP2A TCs Risk Score (TTRS) (left). Heatmap displaying the distribution of prognosis-related genes in the high TTRS group and the low TTRS group (right). **(E)** Forest plot presenting the outcomes of multivariate Cox analysis for clinical factors and risk scores in the training cohort. **(F)** Nomogram model constructed based on the TOP2A TCs Risk Score (TTRS), incorporating race, age, and grade. **(G)** ROC curves evaluating the prediction sensitivity of the nomogram model through the analysis of AUC scores. **(H)** Boxplot displaying the C-index of the AUC at 1, 3, and 5 years. **(I)** Scatter plots and heatmaps showing the pairwise correlations among four prognosis-related genes, OS, and TOP2A TCs Risk Score (TTRS). **(J)** Ridge plots and boxplots illustrating the expression levels of four prognosis-associated genes in groups with high and low TTRS. **(K)** Boxplots displaying the expression levels of four predictive genes in high and low Age groups. **P < 0.01 and ***P < 0.001; ns indicated no significant difference.

Among the four ovarian cancer subgroups identified, the C2 TOP2A+ TCs were potentially at the initial stage of differentiation and had not received neoadjuvant treatment. Consequently, we developed and validated a prognostic risk model for this subgroup. Univariate Cox analysis was performed on the top 100 marker genes within the C2 TOP2A+ TCs subgroup. To address multicollinearity, LASSO regression analysis was conducted with the optimal lambda.min 0.003. The results revealed that four genes were significantly associated with prognosis in the training cohort (**Figure 9A**). Subsequently, the coefficients were determined through multivariate Cox analysis, and the TOP2A TCs Risk Score (TTRS) was calculated for each sample. Based on the median TTRS, the training cohort was divided into the high TTRS group (High TOP2A TCs Risk Score Group) and the low TTRS group (Low TOP2A TCs Risk Score Group). Survival curves, utilizing the Kaplan-Meier method, were plotted for these groups (**Figure 9B**). As anticipated, the high TTRS group exhibited worse prognosis (P < 0.0001). The ROC curve demonstrated that the established prognostic risk model displayed elevated sensitivity and specificity, with corresponding AUC values of 0.595 for 1 year, 0.595 for 3 years, and 0.648 for 5 years (**Figure 9C**). The high and low TTRS groups, as well as the survival and mortality status over time, were depicted in (**Figure 9D**, left). Furthermore, the expression levels of the four prognostic-related genes (UBB, SNRPD1, HSPE1, and HMGB3) in the high TTRS and low TTRS groups were shown (**Figure 9D**, right).

Multivariate Cox analysis was conducted to assess the independent predictive ability of TTRS for overall survival (OS) in the TCGA database (**Figure 9E**). A nomogram was generated, incorporating the TOP2A TCs Risk Score (TTRS), age categories (high and low), race (American Indian or Alaska native, Asian, black or African American, and white), and tumor grades (G2, G3, GB, and GX), to forecast the overall survival rate at 1, 3, and 5 years for the training cohort (**Figure 9F**). The sensitivity and specificity of the nomogram model were evaluated, resulting in AUC values of 0.62, 0.64, and 0.66 for 1 year, 3 years, and 5 years, respectively (**Figure 9G**). The concordance index (c-index) results indicated that the nomogram model exhibited strong predictive capabilities (**Figure 9H**). The correlation between the four prognostic-related genes and OS, as well as TTRS, was analyzed and visualized using scatter plots and heatmaps (**Figure 9I**).

Furthermore, we examined the distribution of the four genes among high and low TTRS categories, as illustrated in **Figure 9J**. Additionally, the expression differences of the four prognostic-related genes in different age groups were analyzed and presented using boxplots. There were no statistical differences observed for the SNRPD1 and HSPE1 genes, but the UBB and HMGB3 genes exhibited higher expression in the Low Age Group (**Figure 9K**).

### Immune infiltration analysis

**Figure 10:**
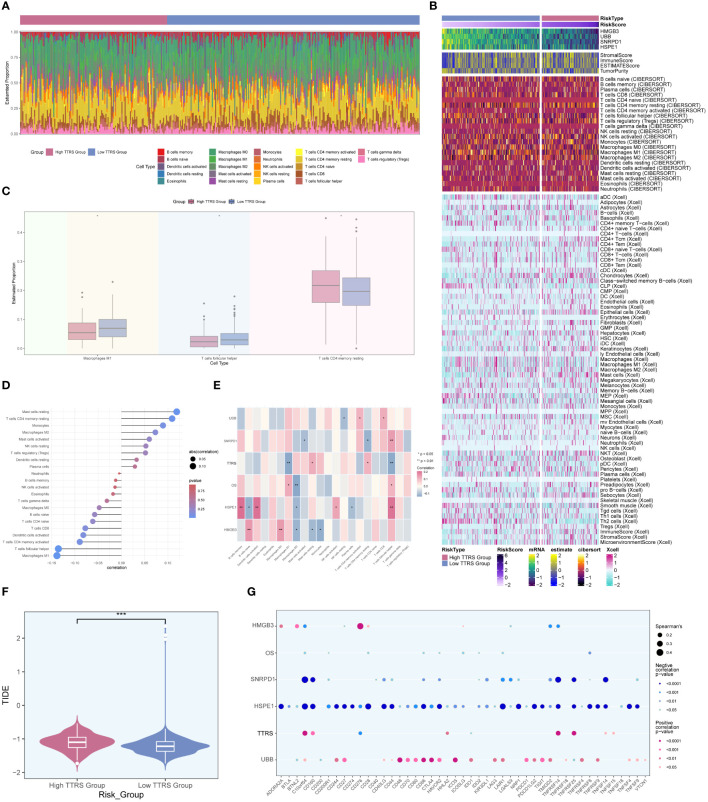
Analysis of immune infiltration in high and low TTRS groups. **(A)** Stacked bar chart displaying the distribution of 22 types of immune infiltrating cells in the high TTRS group and the low TTRS group. **(B)** Heatmap showing the expression of immune infiltrating cells in the high TTRS group and the low TTRS group. **(C)** Boxplot illustrating variations in the levels of Macrophages M1, T cells follicular helper, and T cells CD4 memory resting between the high TTRS group and the low TTRS group. **(D)** Lollipop chart displaying the results of the correlation analysis between immune-infiltrating cells and the TTRS (TOP2A TCs Risk Score). **(E)** Heatmap displaying the relationship between immune cell infiltration, prognosis-related genes, overall survival (OS), and the risk score of TOP2A tumor cells (TTRS). **(F)** Violin showing the difference in the TIDE value between the high TTRS group and the low TTRS group. **(G)** Bubble chart illustrating the relationship between immune checkpoint genes, prognosis-related genes, OS, and the TTRS. *P < 0.05; **P < 0.01; and ***P < 0.001; ns indicated no significant difference.

In order to investigate the heterogeneity between the high TTRS group and the low TTRS group in more depth, we conducted a comprehensive analysis of their tumor immune microenvironment. The distribution of 22 distinct immune-infiltrating cell types in both groups was graphically represented in **Figure 10A**. Subsequently, the CIBERSORT and Xcell algorithms were employed to estimate the proportions of immune infiltrating cells in the high TTRS and low TTRS groups, as shown in **Figure 10B**. Significant disparities were observed in the distribution of immune infiltrating cells between the two groups. Notably, the low TTRS group exhibited elevated levels of macrophage M1 and T cells follicular helper, while the high TTRS group demonstrated higher expression of T cells CD4 memory resting (**Figure 10C**).

The relationship between immune infiltrating cells and the Risk Score of TOP2A TCs (TTRS) was depicted in **Figure 10D**. T cells follicular helper and macrophages M1 exhibited an inverse correlation with TTRS, whereas mast cells resting and T cells CD4 memory resting showed a positive correlation. Moreover, we examined the relationship between immune infiltrating cells and the four prognosis-related genes, as well as their association with overall survival (OS) and TTRS. Gene HSPE1 demonstrated a negative correlation with B cells naïve, macrophages M2, and plasma cells, while gene HMGB3 exhibited a negative correlation with macrophages M2, mast cells resting, and monocytes (**Figure 10E**). To assess the potential tumor immune evasion, we evaluated the Tumor Immune Dysfunction and Exclusion (TIDE) score in both groups, revealing significant differences between them (**Figure 10F**). The high TTRS group exhibited a higher TIDE score, suggesting a greater likelihood of immune escape and potentially poorer response to immune checkpoint inhibitors (ICIs).

Furthermore, we conducted an in-depth analysis of immune checkpoint-associated genes correlation with the four modeling genes, and their relationship with OS and TTRS. The results displayed in **Figure 10G** indicated that gene HSPE1 displayed a negative correlation with most immune checkpoint-related genes, while gene UBB exhibited a positive correlation with a larger subset of immune checkpoint-related genes.

### Enrichment analysis

**Figure 11:**
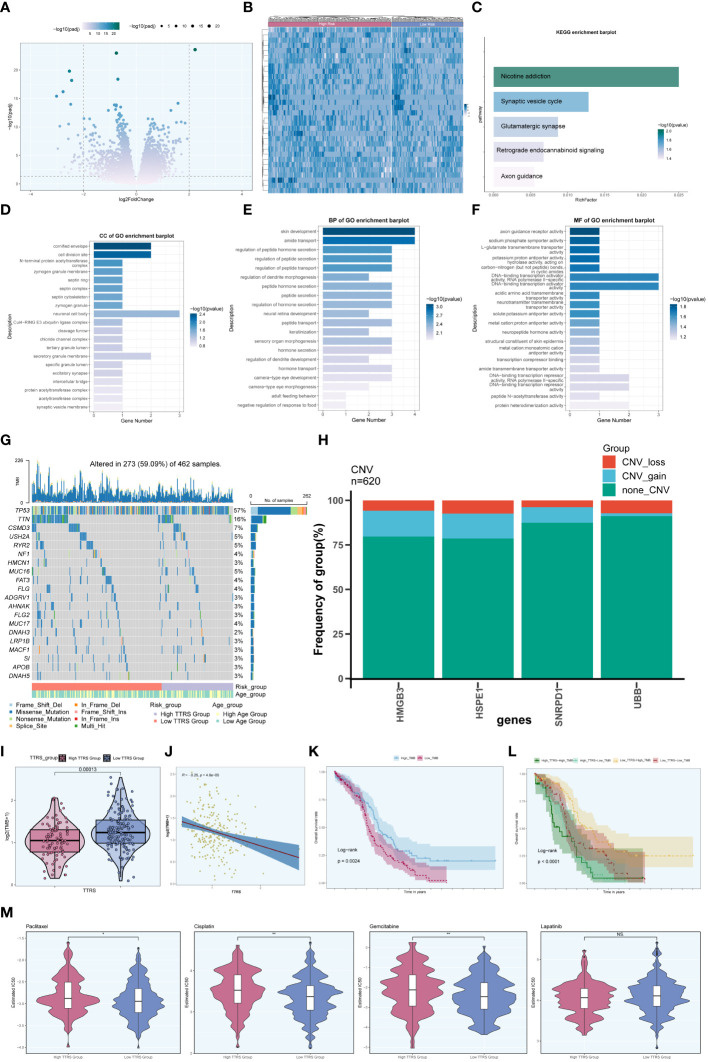
Enrichment analysis. **(A)** Volcano plot presenting DEGs in high and low TTRS groups. **(B)** Heatmap displaying the differential distribution of DEGs in the high TTRS group and the low TTRS group. **(C)** KEGG enrichment analysis results of DEGs. **(D-F)** GOCC, GOBP and GOMF enrichment analysis results of DEGs. **(G)** Mutation waterfall plot depicting the occurrence of mutations in the high and low TTRS groups within the training cohort. The top row illustrated the mutation burden for each sample, while the side column showed the overall percentage of genes in these samples. **(H)** Bar graph showing chromosome copy number variation (CNV) for four genes. Red represented chromosome losses, blue represented chromosome gains, and green represented no chromosome losses or gains. **(I)** Violin plot displaying Tumor Mutation Burden (TMB) values for high and low TTRS groups (P = 0.00013). **(J)** Scatter plot illustrating the relationship between TMB and the risk score of TOP2A TCs, known as TTRS. **(K, L)** Kaplan-Meier survival curves for the high TMB group and the low TMB group, the high TTRS-high TMB group, the high TTRS-low TMB group, the low TTRS-high TMB group, and the low TTRS-low TMB group. **(M)** Violin plots showing the difference in drug sensitivity between high and low TTRS groups. *P < 0.05 and **P < 0.01; ns indicated no significant difference.

To further explore the differences between the high TTRS group and the low TTRS group, we examined the differentially expressed genes (DEGs) between the two groups and explored their enrichment pathways. **Figure 11A** presented the two sets of DEGs identified, and their distribution across the two groups was visualized using a heatmap in **Figure 11B**. Subsequently, we performed enrichment analysis to gain insights into the functional implications of these genes.

In terms of KEGG enrichment analysis, the DEGs were found to be enriched in various pathways, including Nicotine addiction, Synaptic vesicle cycle, Glutamatergic synapse, Retrograde endocannabinoid signaling, and Axon guidance, among others (**Figure 11C**). Additionally, enrichment analysis of DEGs using Gene Ontology Biological Process (GOBP), Cellular Component (GOCC), and Molecular Function (GOMF) revealed intriguing associations. The GOCC enrichment pathway primarily involved the cornified envelope and the cell division site (**Figure 11D**). Furthermore, the GOBP analysis indicated associations with skin development and amide transport (**Figure 11E**), while the GOMF findings suggested a connection to DNA binding transcription activator function, specifically RNA polymerase II, among others (**Figure 11F**).

Moreover, we investigated the frequency of somatic genetic mutations in the training cohort and identified the top 20 genes with the highest mutation rates (**Figure 11G**). TP53 was found to have the highest mutation rate among all genes. Furthermore, we analyzed the chromosome copy number variation (CNV) of the four prognosis-related genes, revealing frequent CNV gain and CNV loss events across all genes, particularly for HMGB3 and HSPE1 (**Figure 11H**).

Furthermore, we computed the Tumor Mutation Burden (TMB) values for both groups, and a violin plot visualized the distribution of these values (**Figure 11I**). Notably, the low TTRS group demonstrated a higher TMB value. Additionally, there was a negative correlation observed between TMB value and TTRS (**Figure 11J**), suggesting a potential relationship between these variables. Based on the median TMB value, the training cohort was stratified into high TMB group and low TMB group. Combining the TTRS groups with the TMB groups, the overall survival analyses revealed statistically significant differences among the groups (**Figures 11K, L**). Interestingly, a higher tumor mutational burden (TMB) was associated with a more favorable prognosis (P = 0.0024) (**Figure 11K**), and the High Risk-High TMB group exhibited the lowest survival rate compared to the other three groups (**Figure 11L**).

### Drug sensitivity analysis of high and low TTRS groups

We assessed the sensitivity of the high TTRS group and the low TTRS group to various chemotherapeutic drugs by calculating their half-maximal inhibitory concentration (IC50) values (**Figure 11M**). The estimated IC50 levels for Paclitaxel, Cisplatin, and Gemcitabine were higher in the high TTRS group, while Lapatinib showed no significant difference between the groups. These findings may inform the selection of more suitable treatment measures based on the differences in drug sensitivity among different groups.

### Experimental result

**Figure 12:**
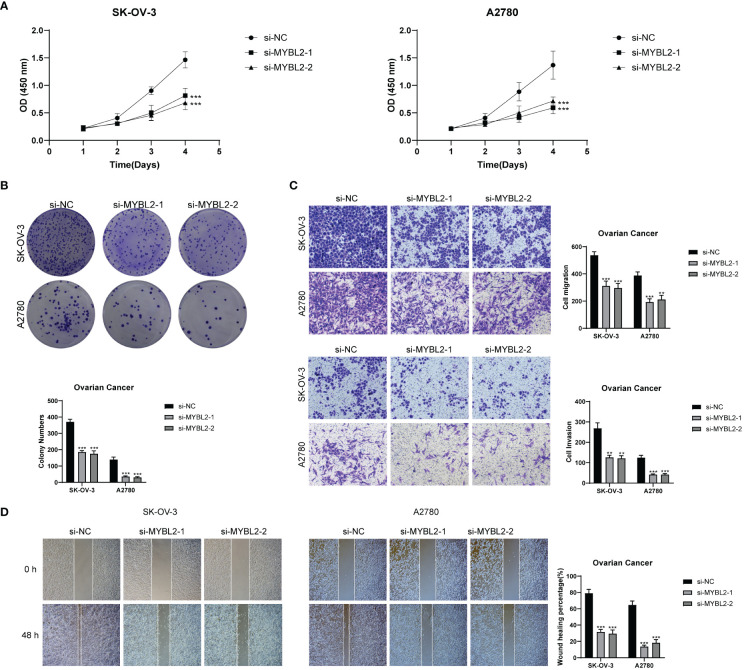
*In Vitro* Experimental Validation. **(A)** CCK-8 assay showing a notable reduction in cell viability in the SK-OV-3 and A2780 cell lines after MYBL2 knockdown. **(B)** Plate cloning assay demonstrating a significant decrease in cell colony counts following MYBL2 knockdown compared to the negative control group. **(C)** Transwell assay displaying a significant reduction in cell migration and invasion in both SK-OV-3 and A2780 cell lines after MYBL2 knockdown. **(D)** The wound healing assay revealed a significant decrease in the migration rate of SK-OV-3 and A2780 cells with MYBL2 knockdown. **P < 0.01 and ***P < 0.001.

MYBL2 wielded a momentous influence on the proliferation and migration of ovarian cancer cell lines. To delve deeper into the functional significance of MYBL2 in ovarian cancer cell lines, we delved into the repercussions of MYBL2 knockdown on two particular cell lines, namely SK-OV-3 and A2780. The outcomes of the CCK-8 experiment highlighted a noteworthy reduction in the proliferation prowess of both cell lines within the MYBL2 knockdown group, when juxtaposed with the control group. (**Figure 12A**). The plate cloning experiment revealed a significant reduction in the growth potential of SK-OV-3 and A2780 cells upon MYBL2 knockdown (**Figure 12B**). The transwell experiment corroborated the inhibitory impact of MYBL2 knockdown on cellular migration and invasion. This was substantiated by the notable reduction in the abundance of cells traversing the lower chamber (**Figure 12C**). The wound healing assay results indicated a statistically significant decrease in cell migration rate in the MYBL2 knockdown group compared to the control group (**Figure 12D**). Therefore, it can be concluded that MYBL2 knockdown significantly suppresses the proliferation and migration abilities of ovarian cancer cells.

## Discussion

Ovarian cancer, a prevalent malignancy of the female reproductive system, often presents with ascites, the accumulation of fluid in the abdomen (2). Late detection and limited treatment options contribute to the high mortality rates associated with this disease. The standard treatment modalities for ovarian cancer include surgery and chemotherapy, while neoadjuvant chemotherapy (NACT) has emerged as a promising therapeutic approach for advanced cases (6). Recently, single-cell multi-omics technologies have provided powerful tools for studying tumor immunology (56). In this study, we utilized single-cell RNA sequencing to investigate malignant epithelial cells associated with omental metastasis in ovarian cancer patients and examined the impact of neoadjuvant therapy on distinct subgroups of malignant epithelial cells.

To unravel the intratumoral heterogeneity of malignant epithelial cells in omental metastasis, we further categorized the acquired cells into four subtypes: C0 CAND2+ TCs, C1 UBB+ TCs, C2 TOP2A+ TCs, and C3 TEX41+ TCs, based on their respective marker genes. The analysis of these subgroups revealed that C2 TOP2A+ TCs exhibited a significantly higher proportion of cells in the G2M phase, indicating their robust proliferative capacity. Notably, the majority of C2 TOP2A+ TCs originated from patients who did not receive neoadjuvant therapy, suggesting a potential interplay between this subgroup and therapeutic intervention. Moreover, integrated CytoTRACE analysis revealed a higher cytotrace score for the C2 subgroup, and slingshot pseudotime analysis indicated an early stage of differentiation. Therefore, the C2 TOP2A+ TCs subgroup appears to be intricately involved in the progression of ovarian cancer. The gene TOP2A, after which this subgroup is named, has been significantly associated with tumor occurrence, invasiveness, therapeutic response, and prognosis (57). Its crucial role in cell division, particularly in the condensation and separation of chromosomes during mitosis (58), makes it a promising target. Prior studies have shown that mutations in the TOP2A gene have a significant effect on the outlook for individuals with ovarian cancer (59), with increased levels of TOP2A protein expression observed in ovarian cancer tissues (55). It was speculated that neoadjuvant chemotherapy (NACT) could promote the transition of the highly proliferative C2 TOP2A+ TCs subgroup to those with lower proliferative capacity. Therefore, NACT could potentially yield better outcomes in ovarian cancer patients exhibiting high TOP2A expression.

Enrichment analysis of the C2 TOP2A+ TCs subgroup revealed a strong correlation with processes such as chromosome segregation, mitotic nuclear division, nuclear chromosome segregation, sister chromatid segregation, and nuclear division. These findings underscore the connection between this subgroup and the genetic material and division processes of ovarian cancer cells, highlighting the importance of investigating the C2 TOP2A+ TCs subgroup as a distinct research entity.

Further analysis of transcription factors across different subgroups identified key transcription factors for each subgroup: PBX1 for C0 CAND2+ TCs, CEBPG for C1 UBB+ TCs, MYBL2 for C2 TOP2A+ TCs, and FOXO1 for C3 TEX41+ TCs. Remarkably, MYBL2 emerged as the top transcription factor in the critical C2 subgroup, which exhibited a higher proportion of cells in the G2M phase and predominantly originated from patients who did not receive neoadjuvant therapy. These findings suggest the potential influence of neoadjuvant therapy on MYBL2 expression. MYBL2, a proto-oncogene belonging to the MYB family, plays a crucial role in tumor development and progression, being commonly upregulated in various cancer types and associated with poor patient outcomes (52). Previous studies have shown that the transcription factor MYBL plays a role in the growth and spread of bladder cancer, while reducing MYBL2 levels effectively inhibits the proliferation and spread of bladder cancer cells, resulting in a halt in the G2 phase (53). However, the specific biological functions of MYBL2 in ovarian cancer remain to be elucidated. Prior studies have shown the presence of the MYBL2-ATAD2 signaling pathway in individuals with OC, emphasizing its importance in regulating the growth of ovarian cancer cells (54). MYBL2 activated CCL2 transcription, inducing TAM recruitment and M2-like polarization *in vitro*. The MYBL2-CCL2 axis promoted tumor progression in ovarian cancer by inducing immunosuppressive macrophages (60). This study provided experimental evidence that the inhibition of MYBL2 through knockdown significantly suppresses the proliferation and migratory capacity of ovarian cancer cells. Therefore, MYBL2 could be a promising focus in relation to ovarian cancer.

To assess the impact of the C2 TOP2A+ TCs subgroup on the progression of ovarian cancer and patient outcomes, we developed a novel predictive model, the TOP2A TCs Risk Score (TTRS), through multivariate Cox analysis. This score allows the division of the training cohort into the High TTRS Group and the Low TTRS Group based on the median TTRS value, with the High TTRS Group exhibiting a poorer prognosis. Immune checkpoints play a vital role in regulating immune responses to prevent excessive reactions. Tumor cells often upregulate immune checkpoints as a mechanism to dampen local immune responses and evade immune surveillance (61).The presence of inhibitory immune checkpoints may contribute to the immunosuppressive nature of the tumor immune microenvironment (TME). The TME exerts a significant influence on tumor progression and treatment efficacy (62, 63).Examining the tumor immune environment of the High and Low TTRS Groups, we found that the High TTRS Group showed a higher presence of resting CD4 memory T cells, while the Low TTRS Group had elevated levels of M1 macrophages and follicular helper T cells. Cancer cells can activate immune checkpoint mechanisms to evade immune recognition. In the high TTRS group, the tumor immune dysfunction and exclusion (TIDE) score was higher, indicating an increased potential for immune evasion in the high TTRS ovarian cancer group, which may lead to a diminished effectiveness of immunotherapy. Additionally, drug sensitivity analysis revealed lower estimated IC50 values for Paclitaxel, Cisplatin, and Gemcitabine in the Low TTRS Group, indicating higher sensitivity to these drugs. However, previous studies have shown that NACT can lead to platinum resistance (8). Therefore, while individuals in the Low TTRS Group may initially respond well to NACT, the potential for developing resistance to platinum-based therapies like Cisplatin should be carefully considered.

It is important to acknowledge the limitations of this study. Firstly, the sample size was relatively small, focusing specifically on the single-cell data of a subgroup of ovarian cancer patients. This limited sample size may affect the statistical power of the findings and their generalizability. Secondly, the study solely relied on single-cell sequencing and transcriptomic analysis. This reliance excludes other layers of biological information that might be crucial for a comprehensive understanding of the disease. Future investigations should involve multicenter studies with larger sample sizes to validate the roles of MYBL2 and the TOP2A TCs Risk Score in ovarian cancer. Additionally, expanding the study to include proteomics and metabolomics approaches could provide a more holistic view of the functional characteristics of specific subgroups and key molecules. This integration could yield deeper insights into the mechanisms underlying ovarian cancer and enhance the potential for developing targeted diagnostic and therapeutic strategies.

Our research focused on the diversity within malignant epithelial cells in metastatic ovarian cancer at the individual cell level, revealing the significance of TOP2A and MYBL2 in this type of cancer. Additionally, we identified prognostically relevant genes, with a higher TOP2A TCs Risk Score (TTRS) indicating a poorer prognosis. The studies help improve comprehension of the development to medication in OC, providing new opportunities for predicting and diagnosing the cancers.

## Conclusion

This study delved deeply into the intratumoral diversity within malignant epithelial cells in ovarian cancer at the single-cell level. The highly proliferative C2 TOP2A+ TCs subgroup likely played a key role in regulating ovarian cancer cell proliferation through related biological processes. This subgroup was highly sensitive to neoadjuvant chemotherapy, which promoted its transition to other subgroups with lower proliferative capacity. The results showed that MYBL2 in C2 TOP2A + TC subgroup was involved in the development and progression of ovarian cancer. The TOP2A TCs Risk Score (TTRS) offers a promising prognostic model that can guide future therapeutic strategies and prognostic assessments. Further research efforts should focus on validating the roles of MYBL2 and the TTRS in larger cohort studies while integrating complementary proteomic and metabolomic approaches to gain a more comprehensive understanding of ovarian cancer biology. These findings offer new insights for future therapeutic strategies and prognostic assessments in ovarian cancer.

## Data availability statement

The original contributions presented in the study are included in the article/**Supplementary Material**. Further inquiries can be directed to the corresponding author.

## Author contributions

WS: Data curation, Methodology, Writing – original draft. ZL: Data curation, Methodology, Software, Supervision, Visualization, Writing – review & editing. ZX: Methodology, Validation, Writing – review & editing. FZ: Methodology, Supervision, Visualization, Writing – review & editing. JX: Methodology, Supervision, Writing – review & editing. XL: Methodology, Supervision, Writing – review & editing. PC: Resources, Supervision, Writing – review & editing.

## Supplementary material

Supplementary Figure 1 Malignant cell identification. **(A)** Hierarchical heatmap displaying the extent of copy number variation (CNV) in epithelial cells, identifying high-level CNV as indicative of malignancy. Red represented gain, and blue represented loss. **(B)** Hierarchical heatmap illustrating the differences in copy number variation (CNV) among subgroups and assessing the variability within subgroups. Using ECs as a reference, red represented gain, and blue represented loss.
