# Supplementary figures and images for: Single-cell RNA sequencing reveals that MYBL2 in malignant epithelial cells is involved in the development and progression of ovarian cancer
Source: Front Immunol. 2024 Jul 29;15:1438198. doi: 10.3389/fimmu.2024.1438198 (PMC11317301; doi:10.3389/fimmu.2024.1438198)

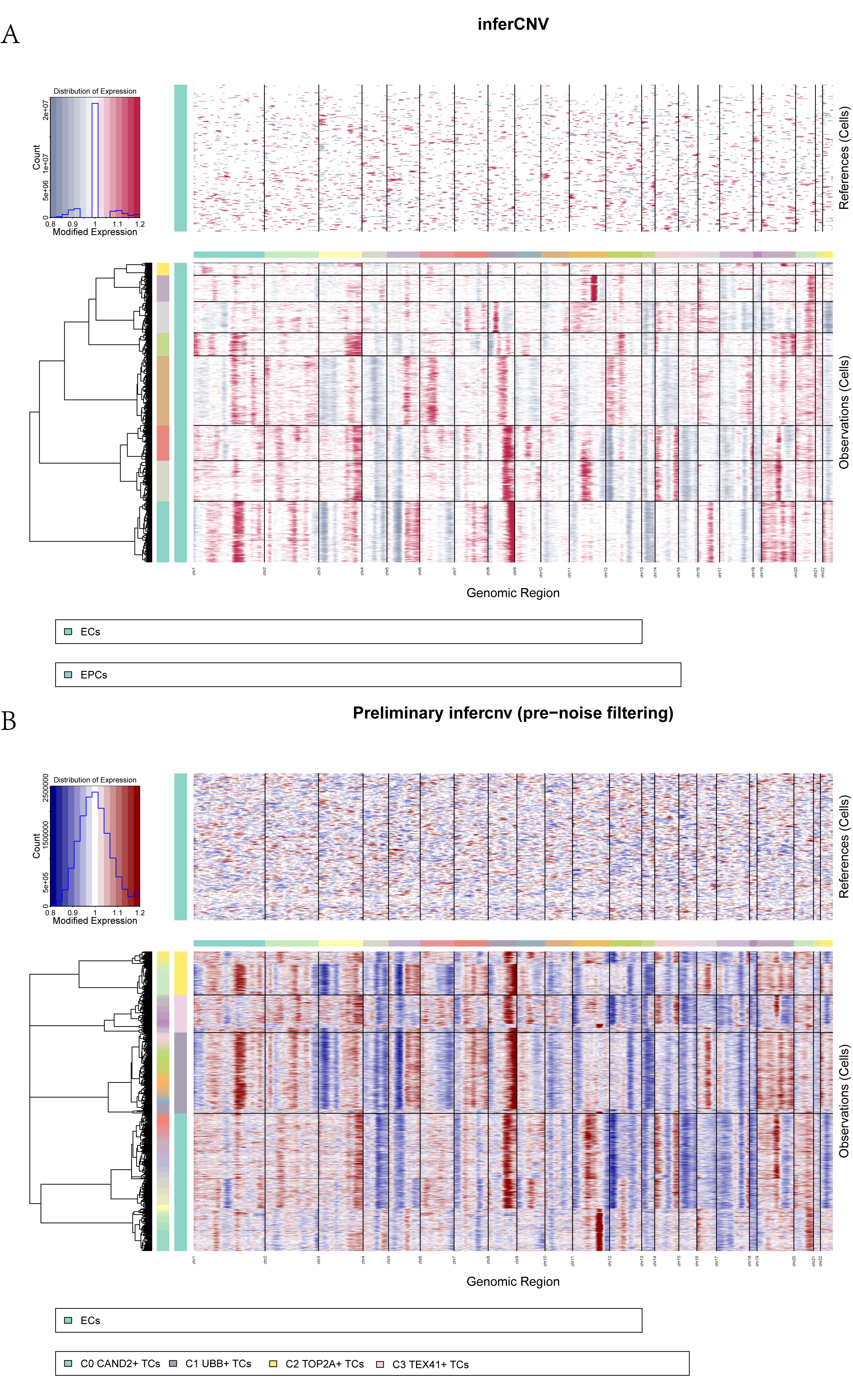

Supplement: Supplementary Figure 1 — Malignant cell identification. (A) Hierarchical heatmap displaying the extent of copy number variation (CNV) in epithelial cells, identifying high-level CNV as indicative of malignancy. Red represented gain, and blue represented loss. (B) Hierarchical heatmap illustrating the differences in copy number variation (CNV) among subgroups and assessing the variability within subgroups. Using ECs as a reference, red represented gain, and blue represented loss. [file Image_1.tif]
